# Comparative Metabolomics Reveals Fungal Conversion of Co-Existing Bacterial Metabolites within a Synthetic *Aspergillus*-*Streptomyces* Community

**DOI:** 10.3390/md19090526

**Published:** 2021-09-19

**Authors:** Yutong Shi, Yihan Ma, Jihua Wei, Yichao Ge, Wei Jiang, Shan He, Xiaodan Wu, Xiaoqin Zhang, Bin Wu

**Affiliations:** 1 Ocean College, Zhejiang University, Zhoushan 321000, China; shiyutong@nbu.edu.cn (Y.S.); mayihan@zju.edu.cn (Y.M.); 11834030@zju.edu.cn (J.W.); geyichao@zju.edu.cn (Y.G.); jw6912@163.com (W.J.); zxqymz881018@163.com (X.Z.); 2 Li Dak Sum Yip Yio Chin Kenneth Li Marine Biopharmaceutical Research Center, College of Food and Pharmaceutical Sciences, Ningbo University, Ningbo 315800, China; heshan@nbu.edu.cn; 3 Center of Analysis, Zhejiang University, Hangzhou 310058, China; fxzhxx@zju.edu.cn

**Keywords:** secondary metabolites, co-occurring microorganisms, fungal–bacterial community, hydrothermal vent, notoamides

## Abstract

In nature, secondary metabolites have been proven to be the essential communication media between co-occurring microorganisms and to influence their relationship with each other. In this study, we conducted a metabolomics survey of the secondary metabolites of an artificial co-culture related to a hydrothermal vent fungal–bacterial community comprising *Aspergillus sclerotiorum* and *Streptomyces* and their reciprocal relationship. The fungal strain was found to increase the secretion of notoamides and the compound cyclo(Pro-Trp) produced by the actinomycetes strain was discovered to be the responsible molecule. This led to the hypothesis that the fungi transformed cyclo(Pro-Trp) synthesized by the actinomycetes as the biosynthetic precursors of notoamides in the chemical communication. Further analysis showed *Streptomyces* sp. WU20 was efficient in transforming amino acids into cyclo(Pro-Trp) and adding tryptophan as well as proline into the chemical communication enhanced the induction of the notoamide accumulation. Thus, we propose that the microbial transformation during the synthetic metabolically-mediated chemical communication might be a promising means of speeding up the discovery of novel bioactive molecules. The objective of this research was to clarify the mechanism of microbial transformation for the chemical communication. Besides, this research also highlights the utility of mass spectrometry-based metabolomics as an effective tool in the direct biochemical analysis of community metabolites.

## 1. Introduction

Natural products (NPs) continue to play a significant role in the discovery and development of new drugs, and this is one of the main motivations for continuing research in the field. Microorganisms have been proven to possess the rich genetic potential to synthesize structurally and functionally diverse secondary metabolites (SMs) [1,2,3]. However, the number of observed NPs produced in standard laboratory conditions is in contradiction to the number of NPs gene clusters present in sequenced genomes (the former being much less) [4,5]. To overcome such limitations, various culture-based approaches have been exploited to expand chemical diversity, such as the one strain many compounds (OSMAC) approach [6], co-cultures [7], epigenetic modification [8], etc. Among these strategies, microbial co-culture, consisting of multiple microorganisms together in the same confined environment has attracted tremendous attention. It allows the microorganisms to initiate chemical interactions and such biological stimuli result in the biosynthesis of diverse NPs by unlocking cryptic pathway expression [9].

Many synthetic microbial communities generated by co-culture techniques are composed of two or more unrelated strains with different backgrounds, which rarely mimic natural ecological interactions [10]. As the “chemical language” in natural microbial communities, SMs are often involved in interspecies interactions or appear as a result of them, such as in representative events including chemical attack and defense, expansion of the chemical space, resource competition, antiviral infection, predation and anti-predator behavior, and chromosome remodeling [11]. For instance, metabolite exchanges, metabolization and/or detoxification of defense molecules have been deciphered in co-culture systems utilizing the cohabitant strains that originally evolved in the same micro-ecological environment [12,13].

In co-culture experiments, another challenging goal is to identify secondary metabolites that are either produced de novo or are up- or downregulated upon interspecies competition [14]. Mass spectrometric (MS) detection generates large datasets, and in addition to the major metabolites, minor constituents can be sensitively and selectively detected [15]. Recently, advances in MS-based comparative metabolomics analyses have been coupled to enable high-throughput analysis of secondary metabolites for the comparison of chemical clues in cellular processes, and intra-organ and inter-organ communication [16]. Correspondingly, metabolomics information helps researchers to gain insights into how microorganisms respond to the interspecies chemical communication and may also provide a means of investigating the biological mechanisms of newly isolated NPs, based on the metabolic changes engendered within the synthetic communities [17].

In this study, we performed a metabolomics survey of the secondary metabolites of an artificial co-culture related to a hydrothermal vent fungal–bacterial community comprising *Aspergillus sclerotiorum* DX9 and *Streptomyces* sp. WU20. Both strains were isolated from sulfur-rich sediments around the Kuishantao hydrothermal vent off Taiwan. The fungal strain was found to increase the secretion of notoamides, and cyclo(Pro-Trp) produced by the actinomycetes was discovered to be the responsible molecule, which led to the hypothesis that cyclo(Pro-Trp) secreted by the actinomycetes was the precursor of fungal notoamides in the chemical communication. Further analysis showed that *Streptomyces* sp. WU20 was efficient in transforming amino acids into cyclo(Pro-Trp). Moreover, adding tryptophan and proline into the chemical communication enhanced the induction of the notoamide accumulation. The objective of this research was to clarify the mechanism of microbial transformation for the chemical communications.

## 2. Results and Discussion

### 2.1. Mass Spectrometry-Based Comparative Metabolomics Profiling of Synthetic Multispecies Microbial Communities Locating Major Discriminating Compounds Induced by Metabolically-Mediated Interactions

Microbial species isolated from the same sample are likely to co-evolve through microbial symbiosis in the natural environment [18]. In this study, we aimed to investigate the chemical communication of synthetic multispecies microbial communities, between two microbial strains with the same background, and specifically, a combination of fungal and bacterial strains. Employing our previously-developed co-culturing device (Supplementary Materials, Figure S1) [19,20], we screened nine fungal–bacterial combinations in the microbial culture collection isolated from the same hydrothermal vent sediment sample collected from Kueishantao, Taiwan. A stable fungal–bacterial community composed of *Aspergillus sclerotiorum* DX9 (host strain located outside the guest strain with larger biomass) and *Streptomyces* sp. WU20 (guest strain located inside the host strain with smaller biomass) was successfully generated for metabolomics investigation (Figure S2). The chemical interactions with the bacterial strain *Streptomyces* sp. WU20 were considered as ecological cues to access cryptic biosynthetic pathways of *Aspergillus sclerotiorum* DX9. The major molecules that were uniquely detected in the co-cultures were deduced to be mainly synthesized by the fungal strain as it contributed a larger proportion of the total culture biomass.

**Figure 1:**
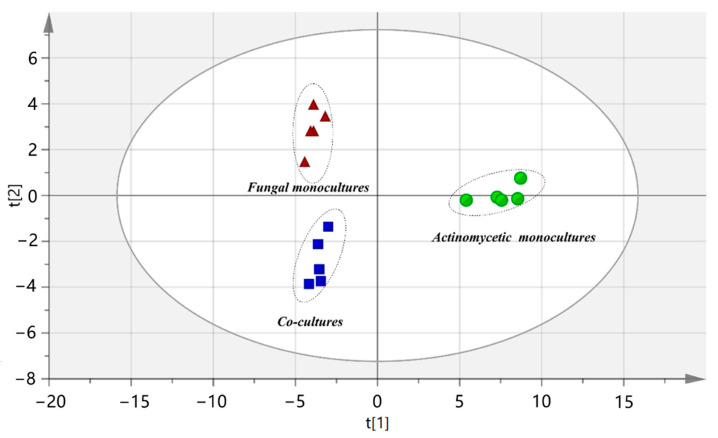
Scores plot based on collected markers for the co-culture group and two related monoculture groups.

A combination of ultra-high-performance liquid chromatography (UHPLC) and high-resolution mass spectrometry (HRMS) was employed to analyze the metabolomes. Due to the fact that many acidic and nonpolar compounds do not ionize well in positive electrospray ionization mode (ESI+), negative electrospray ionization (ESI-) was also applied to cover as many SMs as possible in the investigations [21]. The metabolic profiles, i.e., the presence and abundance of detected features, for both the co-culture and monoculture samples were compared using principal component analysis (PCA) [22]. The PCA result showed the clustering of the samples in three groups, indicating that the co-culture fingerprints did not overlap with the two corresponding monoculture clusters (Figure 1). The separation meant that the datasets contained information that allowed for the discrimination of the chemical composition of the co-cultures from that of the monocultures, implying that chemically-mediated interactions modulated the biosynthetic pathways for the production of secondary metabolites.

Then, orthogonal projections to latent structure coupled with discriminant analysis (OPLS-DA) (including two OPLS-DA models for the comparison of the co-culture with each corresponding monoculture) was utilized to locate mass spectrometric markers exclusively related to the co-culture group [23]. Each model permits the classification of features according to its capacity to separate two groups (the co-culture and one of the pure-strain cultures). In order to select features that are highly specific to the co-culture compared to a single pure-strain culture, the datasets of the co-culture and datasets of a related monoculture are compared in an *S*-plot. In this plot, the *x*-axis denotes the contribution of a marker to the differences in the grouping, and the *y*-axis denotes the confidence of this contribution. Thus, the markers in the lower-left corner are characteristic of the co-culture, whereas the markers in the upper right corner are characteristic of the monoculture.

**Figure 2:**
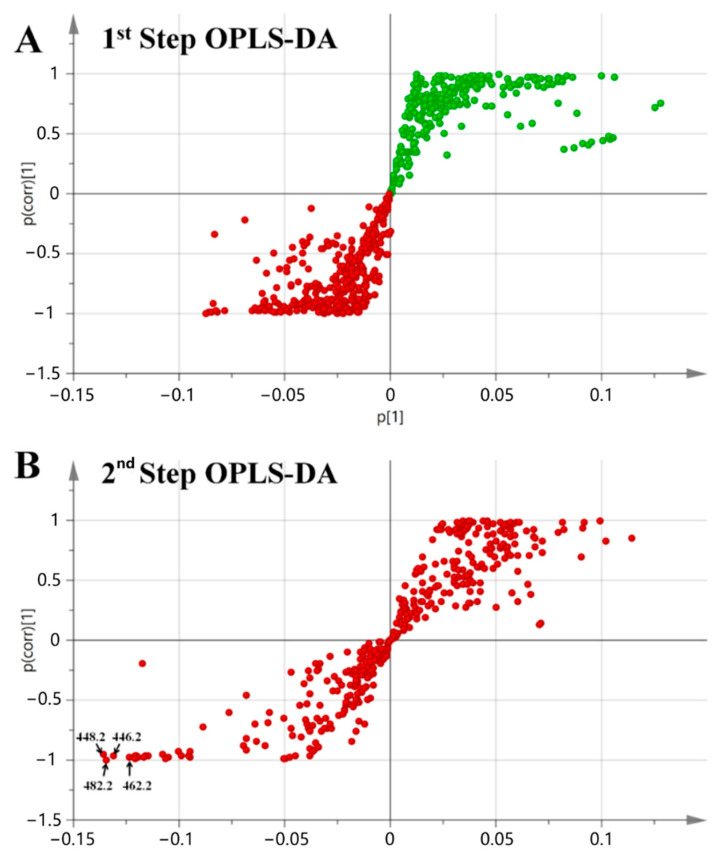
Two-step OPLS-DA for locating major discriminating compounds induced by fungal–bacterial chemical communication. The *S*-plot of the co-culture samples and the bacterial monoculture samples was shown in (**A**). The markers in the first quadrant were excluded as they mainly represented molecules produced by *Streptomyces*. The markers in the third quadrant were then included into the second OPLS-DA (**B**) for the comparison of the co-culture and the fungal monoculture.

In detail, the datasets of both the co-culture and the bacterial monoculture were compared in the first-step *S*-plot (Figure 2A). The green markers in the first quadrant represent the molecules produced by *Streptomyces* sp. WU20 in the bacterial monoculture as well as secondary metabolites secreted by bacteria in the co-culture, which can also result in the discrimination of the co-culture from the fungal monoculture. The red markers shown in the third quadrant are composed of molecules produced by the fungi in both the co-culture and monoculture. Thus, they were then included in the second OPLS-DA (Figure 2B) for the comparison of the co-culture and the fungal monoculture. The markers shown in the first quadrant are characteristic of the *Aspergillus* monoculture representing the secondary metabolites that were downregulated upon chemical interactions with *Streptomyces* in the co-culture. The presence of these markers also indicates shifts in the biosynthetic pathways and metabolic regulation upon chemical communication with bacteria. The markers in the third quadrant represent the secondary metabolites, which were either produced de novo or were upregulated in the co-cultures and the markers in the lower-left corner are the main contributions from the co-cultures to the differences in metabolic profiles. Four markers in the lower-left corner were recognized as four positive ions at *m/z* 448.2, 482.2, 446.2, and 482.2. These four features were set as the four targets with the highest priority in the next step.

### 2.2. Mass Spectral Molecular Networking Facilitating Structural Elucidation of Target Co-Culture-Induced Metabolites

**Figure 3:**
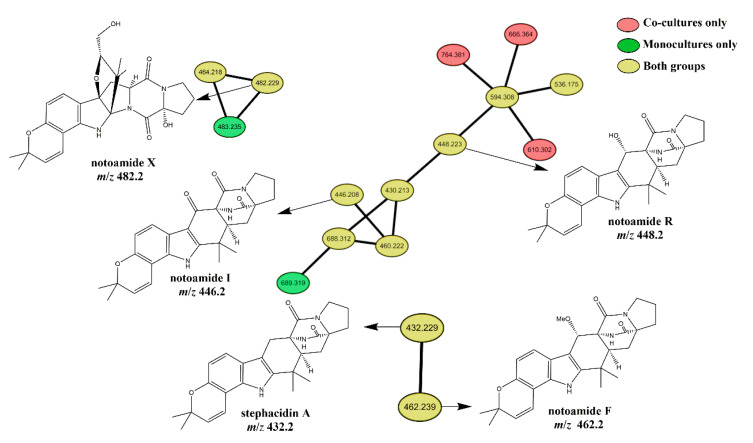
The notoamide-related molecular network generated by a combination of GNPS and Peakview.

**Figure 4:**
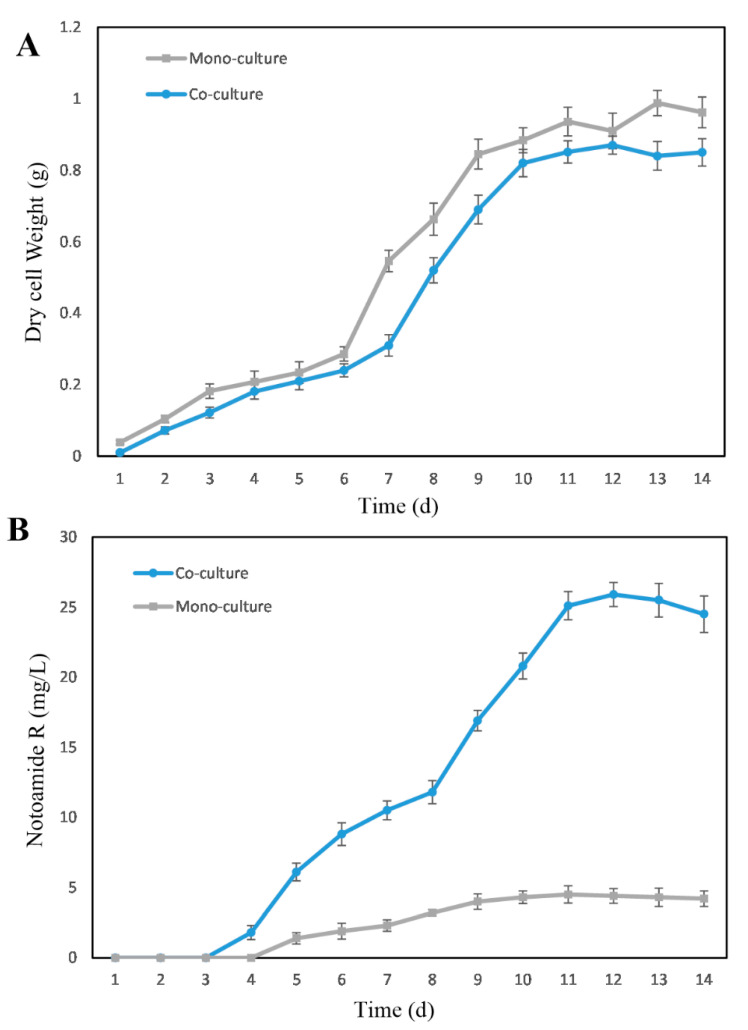
Monitoring of fungal growth (**A**) and notoamide production (**B**) of both the co-cultured *Aspergillus sclerotiorum* (circle) and the corresponding monoculture (square). Values represent means ± standard errors of results from three independent replicates.

As all four markers with the highest priority identified in the discriminant analysis were detected as positive ions, two mass spectrometry datasets belonging to the co-culture group and the fungal monoculture in the positive-ion mode were transferred onto GNPS to generate a comprehensive network (Figure S3). As a result, four targeted features positively-correlated with the co-culture group were present in the network (represented as yellow nodes); these features were detected in both datasets (Figure 3). That is to say, these four molecules were produced under both the co-culture and the monoculture but their concentration in the chemical communication increased when compared to the monocultures. The feature at *m/z* 448.2 in an 11-membered cluster was putatively identified as notoamide R ([M + H]^+^, C_26_H_30_N_3_O_4_), which is a known secondary metabolite [24] recognized in the GNPS molecular library. Another target at *m/z* 446.2 in the same cluster was recognized by the GNPS molecular library as notoamide I [25] ([M + H]^+^, C_26_H_28_N_3_O_4_), which possesses a carbon backbone similar to notoamide R. The marker at *m/z* 462.2 in a two-membered family was matched with notoamide F [25] ([M + H]^+^, C_26_H_28_N_3_O_4_) and its neighboring yellow node at *m/z* 432.2 was found to be stephacidin A, which also has a similar chemical structure. Besides, the feature at *m/z* 482.2 ([M + H]^+^, calculated for C_26_H_32_N_3_O_6_), was elucidated as a previously undescribed notoamide-type structure (named as notoamide X) by spectroscopic data analyses following the natural product isolation of crude extracts from the large-scale monoculture (for detailed structure elucidation, see the Supplementary Materials). Thus, the four targeted positive ions at *m/z* 448.2, 482.2, 446.2, and 482.2 were presumed to be notoamide R, notoamide X, notoamide I and notoamide F, respectively. Examination of the initial PCA data verified that these four related alkaloids all showed significantly enhanced levels (6-fold, 3-fold, 2.5-fold and 2-fold increase, respectively) in the co-cultures when compared to monoculture controls (Table S2). The production of stephacidin A was distinctly different in the co-cultures and monocultures, with the content in the co-culture group enhanced by a factor up to 1.5 compared with the monocultures. It is worth noting that notoamide B, a chemical isomer of notoamide R at *m/z* 448.2 was detected with a different retention time in both co-cultures and monocultures, whereas there was no significant difference between the two groups. To further investigate the differences between the co-cultured *Aspergillus sclerotiorum* DX9 and the corresponding monoculture, fungal growth and the production of notoamide R were both examined at 24 h intervals (Figure 4). During the total 14 days of monitoring, the co-cultured fungi showed slightly slower growth in the presence of *Streptomyces* sp. WU20 compared to the monoculture. However, despite having less biomass, the co-cultured strain enhanced the biosynthesis of notoamide R up to 7-fold, which also indicated the accumulation of notoamide-type metabolites in the synthetic fungal–bacterial chemical communication.

Besides, the structures of these five alkaloids were closely related, but only two notoamides were found to cluster in the same molecular family. This outcome clearly hinted at the limitation of the GNPS spectral networking algorithm in clustering all the structurally related nodes. Despite populating three different molecular clusters, notoamide X, notoamide I, notoamide F and stephacidin A scored more than 75% similarities (77%, 94%, 93%, and 87%, respectively) to notoamide R, which was set as a reference feature in the similarity scoring function in Peakview (version 2.1, AB SCIEX, Framingham, MA, USA). In a previously described workflow [20], we defined the leads with similarities above 70% as notoamide-similar markers, and linked other molecular clusters where notoamide-similar markers located to the molecular cluster related to notoamide R. All of these five molecules were then included in a newly generated molecular map (Figure 3). Thus, the coverage of notoamide-related features was greatly increased by this combination of GNPS molecular networking and Peakview similarity scoring.

### 2.3. Induction-Effect-Guided Isolation Revealing Cyclo(Pro-Trp) Secreted by Streptomyces as Potential Molecular Inducers of Fungal Secondary Metabolites in the Co-Cultures

**Figure 5:**
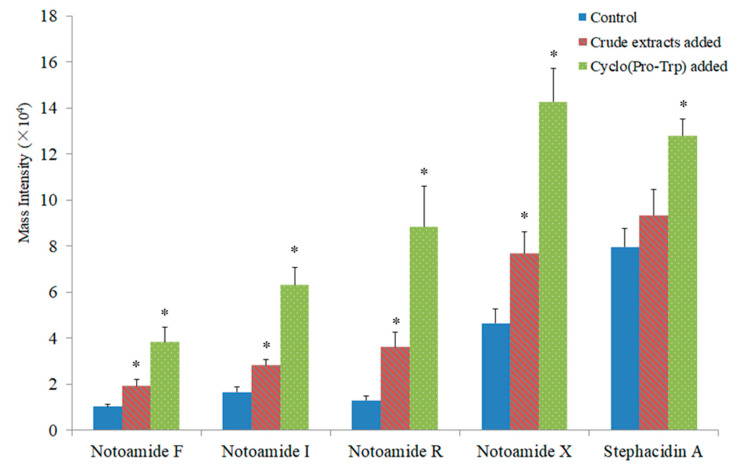
Mass abundances of five notoamide-related molecules produced by *Aspergillus sclerotiorum* fed with bacterial crude extracts or cyclo(Pro-Trp), * *p* < 0.05 vs. control.

Bacterial metabolites in the fungal–bacterial chemical communication were able to induce the metabolic production of co-culturing fungi [19]. To investigate the possible mechanism of the accumulation of these alkaloids, the monoculture of *Aspergillus sclerotiorum* DX9 was fed with 10 mg crude extracts from the monoculture of *Streptomyces*. Interestingly, the production of four target notoamides (notoamide F, I, R and X) by *Aspergillus* fed with such crude extracts increased significantly (*p* < 0.05, Figure 5), compared with the production by untreated fungi. Based on this fact, we confirmed that certain molecules secreted by *Streptomyces* sp. WU20 acting as starter molecules or as elicitors of specific biosynthetic pathways, rather than cell-to-cell contact with or nutrient depletion by bacteria, induced the fungal production of notoamides in the co-culture. To tackle these potential molecular inducers, inductive effects derived from the fungal monocultures fed with fractions of bacterial crude extracts were employed as guides in the isolation and purification of active molecules in the bacterial monocultures, following a strategy similar to bioassay-guided fractionation. Finally, a known cyclodipeptide cyclo(Pro-Trp) (also known as brevianamide F [26]), as one of the major substances in the *Streptomyces* metabolome, was discovered to be the molecule responsible for the accumulation of notoamides. When *Aspergillus sclerotiorum* DX9 was fed with 2 mg of purified cyclo(Pro-Trp) on the fourth day after inoculation, a similar induction of notoamides was observed after 14-day fermentation, with the level of all five notoamide-type structures significantly enhanced (*p* < 0.05, Figure 5).

**Figure 6:**
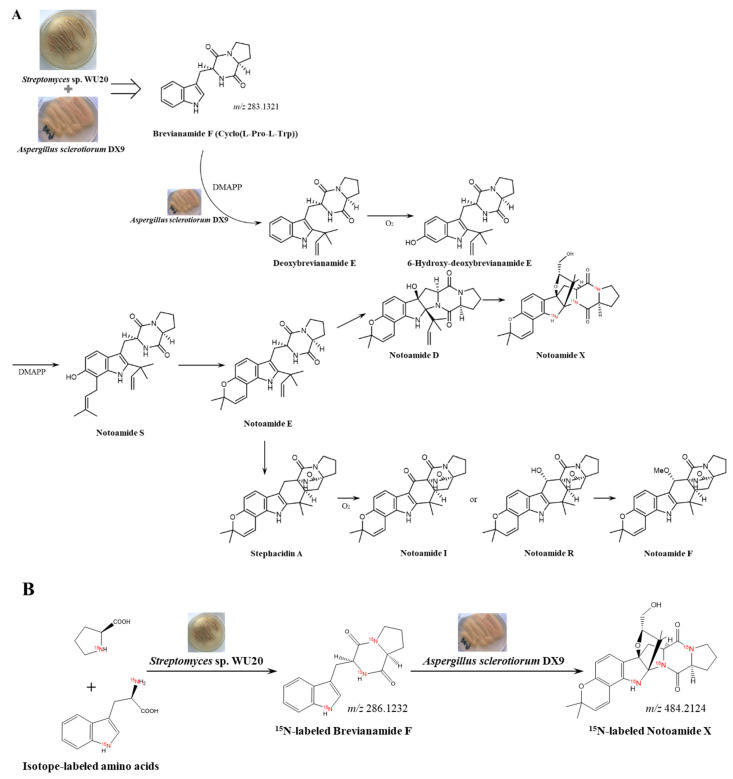
Proposed biosynthetic pathway of notoamides in the fungal–bacterial chemical communication (**A**). Isotopic labeling experiment (**B**) was performed by feeding stable ^15^N-labeled L-proline and L-tryptophan.

Previous research on the biosynthesis of notoamide metabolites from fungal metabolomes has proved that cyclo(Pro-Trp) is an essential precursor in the biosynthetic process of notoamides [27]. In the reported biosynthetic pathway, *Aspergillus sclerotiorum* transformed cyclo(Pro-Trp) to obtain various notoamide structures through multiple steps such as isopentene derivation, oxidation, and intramolecular Diels-Alder reaction (Figure 6A). This meant that *Aspergillus sclerotiorum* had a complete intracellular enzyme system catalyzing the entire synthesis of notoamides by consuming cyclo(Pro-Trp). Therefore, we hypothesized that the accumulation effect of the notoamide-type structure in the chemically-mediated chemical communication might be due to the efficient biotransformation of cyclo(Pro-Trp) by fungi in the interspecies chemical communication. In the artificial microbial community established in the experiment, in addition to its own intracellular synthesis of cyclo(Pro-Trp), the fungi also assimilated the same compound secreted by co-culturing *Streptomyces* as a substrate to synthesize notoamides (Figure 6A), so that the yield of notoamide compounds in the final secondary metabolome was significantly enhanced.

To validate the fungal potential of utilizing the exogenous cyclo(Pro-Trp) in the chemical communication, an isotopic labeling experiment was performed to unveil the microbial transformation (Figure 6B). Stable ^15^N-labeled L-proline and L-tryptophan were incorporated in the 7-day bacterial cultivation. ^15^N-labeled cyclo(Pro-Trp) was then detected in the mass spectrometry-based metabolic profiling of the final fermentation (Figure S14). Then, the EtOAc extracts of the bacterial fermentation were fractionated by HPLC on an Rp-18 column to afford an enriched fraction, which contained ^15^N-labeled cyclo(Pro-Trp) but no labeled amino acids. The target fraction was fed into the fungal monoculture of *Aspergillus sclerotiorum* on the fourth day after inoculation. As a result, ^15^N-labeled notoamides were found to be present in the LC-MS analysis of fungal secondary metabolites (Figure S15), which further supported the above-mentioned fungal assimilation of bacterial metabolites in the co-cultures. However, it is important to note that the growth conditions employed were far from the conditions that the microbial strains have evolved to deal with. Thus, chemical communication here was a highly artificial interaction showing the principle of chemical communications in the synthetic microbial community.

### 2.4. Further Investigation on the Effects of Exogenous Amino Acids on the Metabolism of Both Monocultures and Co-Cultures

**Figure 7:**
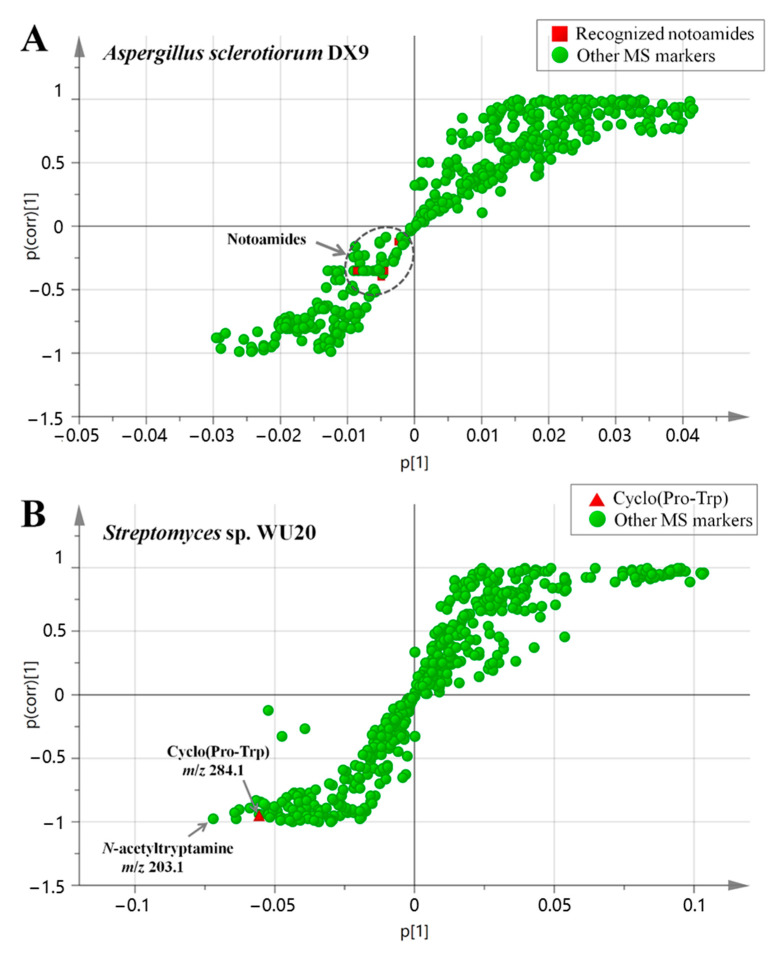
OPLS-DA analysis for the metabolic comparison of Pro-Trp-added cultivations and the untreated cultivation. (**A**) *S*-plot for *Aspergillus sclerotiorum* DX9; (**B**) *S*-plot for *Streptomyces* sp. WU20.

Amino acids are raw compound materials for the biosynthesis of many secondary metabolites, including cyclo(Pro-Trp) and notoamides in this research. However, microbial abilities to convert exogenous amino acids into their metabolites vary with the species [28]. As tryptophan and proline were biogenic precursors for cyclo(Pro-Trp) and notoamide-type skeletons, we monitored the effects of exogenous tryptophan and proline addition on the secretion of cyclo(Pro-Trp) by *Streptomyces* sp. WU20 as well as notoamides by *Aspergillus sclerotiorum* DX9. Tryptophan (200 μM) and proline at the same concentration were added into both fungal and bacterial monocultures. After 14-day fermentation, we used the same LS-MS method as employed in the previous experiments to analyze the secondary metabolites of both the amino-acid-treated cultivations and untreated cultivations. An OPLS-DA model was utilized here to quickly display the contribution made by MS markers to the difference in the two groups and then to evaluate the influence of added amino acids on the metabolic production. The MS features detected in the OPLS-DA analysis were divided into two clusters positively-correlated with the experiment group and control group, respectively. The results for *Aspergillus sclerotiorum* DX9 (Figure 7A) showed that the MS markers on behalf of five notoamide-type compounds were located in the third quadrant, that is, they were all positively correlated with the amino-acid-added group. However, these notoamides and detected cyclo(Pro-Trp) featured in the metabolic files were quite near the origin of the *S-*plot coordinates, indicating that these five notoamide-like molecules contributed little to the difference between groups, and this contribution had a low confidence value. This plot demonstrated the relatively little effect of the amino acid addition on the fungal production of both cyclo(Pro-Trp) and the five recognized alkaloids. So, the strain *Aspergillus sclerotiorum* DX9 possessed a weak ability to convert high concentration of exogenous tryptophan and proline, which might be related to its catalytic efficiency of intracellular cyclic dipeptide synthase (CDPSs).

In the *S*-plot of *Streptomyces* sp. WU20 (Figure 7B), the marker representing cyclo(Pro-Trp) was located in the bottom left corner of the *S*-plot, showing that its yield was highly positively correlated with the group treated with exogenous amino acids. According to the MS abundance of the target cyclic dipeptide in both groups, the production of cyclo(Pro-Trp) in the experimental group was boosted up to 4 times compared to that in the control group. In addition, the molecule at the far left of the *S*-plot was recognized by the molecular library as the known compound *N*-acetyltryptamine, whose secretion increased approximately 6 times compared to in the untreated cultivations. The biosynthetic precursor of *N*-acetyltryptamine was proved to be tryptophan.

**Figure 8:**
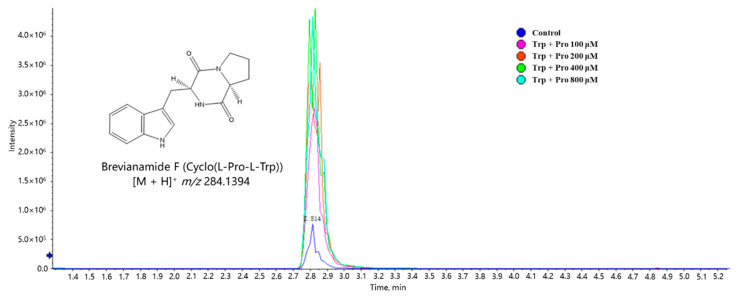
Extracted ion chromatography (EIC) of cyclo(Pro-Trp) from the metabolomes of *Streptomyces* fed with different concentrations of tryptophan and proline.

To further investigate the dose–effect relationship between the addition of tryptophan and proline on the biosynthesis of cyclo(Pro-Trp), the treated culture media consisted of an additional 100, 200, 400, and 800 μM of both tryptophan and proline. The outcome showed that significant cyclo(Pro-Trp) accumulation could be observed with the fortified concentration at 100 μM, but there was no further increase in the yield of target cyclic dipeptide when the level of added amino acid concentration was higher (400 or 800 μM) (Figure 8). This was possibly because the catalytic activities of intracellular CDPSs had approached saturation in the bacterial fermentation treated with high concentrations of tryptophan and proline.

**Figure 9:**
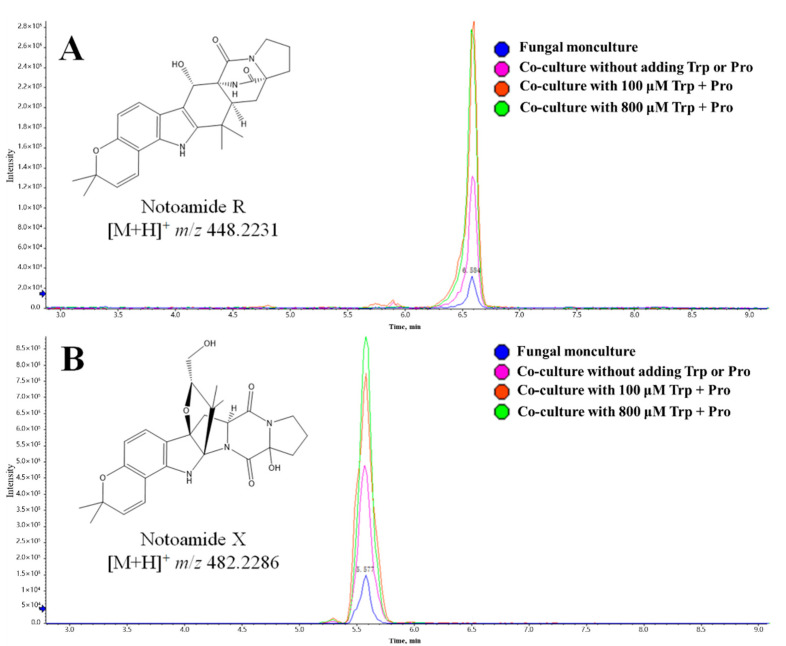
Extracted ion chromatography (EIC) of notoamide R (**A**) and notoamide X (**B**) from the metabolomes of different culture systems.

It was confirmed that *Streptomyces* sp. WU20 can efficiently transform tryptophan and proline in the culture system to increase the secretion of cyclo(Pro-Trp), while exogenous cyclo(Pro-Trp) can induce *Aspergillus sclerotiorum* DX9 to boost the production of notoamide metabolites. Therefore, we examined the effect of exogenous amino acids on the content of notoamide-type structures in the co-culture metabolome. Another OPLS-DA model was established to study the correlational relationship between various MS features and cultivation groups. As a result, two markers representing notoamide R and X were located in the lower left side of the *S*-plot (Figure S4). With the combination of the MS abundance and EIC pattern (Figure 9), we found that the yield of these two compounds could be further significantly enhanced with the addition of exogenous amino acids in the co-culture system. The molecular abundances of notoamide R and X were about 2 times and 1.5 times, respectively, of that in the co-culture group without adding amino acids, and 15 times and 6 times, respectively, of that in the fungal monoculture. However, no significant differences were detected in the contents of the other three notoamide-type structures (notoamide I, notoamide F and stephacidin A) in both synthesized microbial communities. Therefore, we adopted the exogenous addition of tryptophan and proline to successfully realize the further increase in the yield of notoamide-related metabolites from *Aspergillus sclerotiorum* DX9, which accelerates the efficient exploitation and utilization of notoamide-type natural products.

## 3. Materials and Methods

### 3.1. Hydrothermal Vent Microbial Strains Isolation

The strains *Aspergillus sclerotiorum* DX9 and *Streptomyces* sp. WU20 were isolated from hydrothermal vent sediment (121°55′ E, 24°50′ N at depths of 15 m, and the ambient temperature was around 44 °C), collected from Kueishantao, Taiwan. The sediment was homogenized using a blender containing 20 mL sterile natural seawater in aseptic conditions. The resulting homogenate was diluted with sterile seawater (1:5, 1:25, 1:125, 1:625). Under sterile conditions, 200 μL of each dilution was inoculated in quadruplicate on to ISP2, containing 4 g dextrose, 4 g yeast extract, 10 g malt extract, 15 g agar per liter of seawater. The plates were incubated at room temperature for 1–4 weeks until the morphology of the microbes could be distinguished. Each isolate was picked. The pure strains were isolated by reinoculation on agar plates and identified by their 16S rDNA or 18S rDNA sequences for bacterial or fungal strains, respectively.

### 3.2. Culturing Conditions for Co-Cultures

The microbial communities were constructed in 2 L co-culture devices (Shi et al., 2017a). The volume of the culture medium for the host strain and the guest strain was 800 mL and 150 mL, respectively. For the purposes of a minimal impact on the discriminant analysis resulting from medium differences, we used the same culture medium for both strains in the co-culture. The fungi and bacteria were cultured under rocking conditions at 28 °C in co-culture devices containing PDB-LB liquid culture medium (100 g potato lixivium, 10 g dextrose, 5 g yeast extract, 10 g peptone, 10 g NaCl, per liter at pH 7.2) for 14 days. *Aspergillus sclerotiorum* DX9 and *Streptomyces* sp. WU20 were first cultivated in 500-mL Erlenmeyer flasks containing 200 mL PDB-LB medium (pH 7.2) on a rotary shaker at 150 rpm for 3 days before 1 mL of fungal suspension (OD_600_ 0.8) was added to the space outside the dialysis bag and 1 mL *Streptomyces* suspension (OD_600_ 0.8) was added into the dialysis bag. Two co-culturing strains were cultured under rocking conditions at 180 rpm, 28 °C for 14 days. All co-culture experiments were performed in quintuplicate. For the isotopic labeling experiment described in the Results, Streptomyces sp. WU20 was inoculated in the PDB-LB liquid culture media (200 mL × 20) spiked with 400 μM stable ^15^N-labeled L-proline and L-tryptophan (Sigma-Aldrich, St. Louis, MO, USA). The 7-day bacterial fermentation was extracted twice with ethyl acetate to afford 1.4 g crude extracts containing ^15^N-labeled cyclo(Pro-Trp) detected in the LC-MS analysis. Then the EtOAc extracts of the bacterial fermentation were fractionated by HPLC on an Rp-18 column (MeOH-H2O as the mobile phase) to give an enriched fraction B (103 mg), which contained labeled cyclo(Pro-Trp) but no labeled amino acids. This fraction (10 mg per 200 mL) was fed into the fungal monoculture of *Aspergillus sclerotiorum* on the fourth day after inoculation in the same PDB-LB media. The final 14-day fermentation was extracted three times with ethyl acetate to afford the final EtOAc extract for LC-MS analysis.

### 3.3. Metabolomics Sampling and UHPLC-ESI-HR-MS Analysis

The culture broth (200 mL) was extracted three times with 200 mL of ethyl acetate to afford the final EtOAc extract for analysis. The detailed mass spectrometric method is included in the Supplementary Materials. The remaining culture broth of all the co-cultures was also extracted to combine with samples for metabolomics analysis to yield the total crude extract (4680 mg) for further isolation.

### 3.4. Data Processing, Molecular Networking and Multivariate Data Analysis

The HR-MS data were analyzed using Peakview 2.1 (Version 2.1, AB Sciex, Concord, ON, Canada). Molecular networking was conducted at Global Natural Products Social Molecular Networking (GNPS, http://gnps.ucsd.edu/, accessed on 14 September 2021). The mass fragmentation similarity result was calculated using the Similarity Scoring function in Peakview. Statistical analysis of the data was done using SIMCA (version 14.0, Umetrics, Umea, Sweden) for PCA and OPLS-DA analysis.

### 3.5. Induction Effect-Guided Isolation of Cyclo(L-Pro-L-Trp)

*Streptomyces* sp. WU20 (10 L) was cultured in the PDB-LB medium at 180 rpm, 28 °C for 14 days. Then the broth (10 L) was extracted with EtOAc (2 × 10 L), which produced an organic extract (3.2 g). This gummy residue was partitioned between *n*-hexane, dichloromethane, *n*-butanol and H_2_O. Four parts were tested and fed *Aspergillus sclerotiorum* DX9 to check whether they induced notoamide. The dichloromethane part (1.4 g) was confirmed as having induction potential and it was then fractionated by HPLC on an Rp-18 column with MeOH-H_2_O as the mobile phase and yielded nine subfractions (A–I). Subfraction C (174 mg) was tracked by the feeding experiments and further fractionated by HPLC using 45% MeOH/H_2_O as the mobile phase to give four subfractions (C1–C4). Fr. C2 (33 mg) was found to affect the notoamide production of the fungal strain and only contained one main HPLC absorption peak. Then this major peak was purified to yield a pure compound (white powder, 8.2 mg) with induction effects on the fungal production of notoamide-related metabolites. The molecular formula was presumed as C_16_H_17_N_3_O_2_ based on its HRMS data ([M + H]^+^ 284.1395 calculated for C_16_H_18_N_3_O_2_, [M − H]^−^ 282.1248 calculated for C_16_H_16_N_3_O_2_). The chemical structure of this compound was finally elucidated as cyclo(L-Pro-L-Trp) by comparing its 1D NMR data with those in the literature.

## 4. Conclusions

In summary, we performed a metabolomics survey of the secondary metabolites of an artificial co-culture related to a hydrothermal vent fungal–bacterial community comprising *Aspergillus sclerotiorum* DX9 and *Streptomyces* sp. WU20. Both strains were isolated from sulfur-rich sediments around the Kuishantao hydrothermal vent off Taiwan. The fungal strain was found to increase the secretion of notoamides and cyclo(Pro-Trp) produced by the actinomycetes was discovered to be the responsible molecule, which led to the hypothesis that the fungi transformed cyclo(Pro-Trp) synthesized by the actinomycetes as the biosynthetic precursors of notoamides in the chemical communication. Further analysis showed *Streptomyces* sp. WU20 was efficient in transforming amino acids into cyclo(Pro-Trp) and adding tryptophan as well as proline into the chemical communication enhanced the induction of the notoamide accumulation. Thus, we propose that such metabolically-mediated interactions might play a fundamental role in microbial ecosystem functioning and could serve as a potential trigger for unveiling bioactive natural products. Especially, the microbial potential to convert metabolites secreted by co-existing microorganisms could be utilized in the exploration of novel antibiotics and other medically relevant natural products. Besides, this study highlights the utility of MS-based metabolomics as an effective tool in the direct biochemical analysis of community metabolism.

## Data Availability

Part of the data presented in this study are available in Supplementary Material here. The remaining data presented in this study are available on request from the corresponding author.

## Supplementary Materials

The following are available online at https://www.mdpi.com/article/10.3390/md19090526/s1, Figures S1–S15; Tables S1–S2. FigureS1: Establishment of a previously-described stationary co-culture device for culturing of multispecies strains that are physically separated but can exchange small molecules. Figure S2: A synthetic fungal-bacterial community composed of Aspergillus sclerotiorum DX9 and Streptomyces sp. WU20 generated in a co-culture device. Figure S3: Molecular networking of the samples derived from the co-culture and the corresponding fungal monoculture. Figure S4: OPLS-DA analysis for the metabolic comparison of Pro-Trp-added co-cultivations and untreated co-cultivations. Figure S5: ^1^H NMR spectrum (500 MHz) of purified cyclo(Pro-Trp) in CD3OD. Figure S6: ^13^C NMR spectrum (125 MHz) of purified cyclo(Pro-Trp) in CD3OD. Figure S7: ^1^H NMR spectrum (500 MHz) of purified notoamide X in CD3OD. Figure S8: ^13^C NMR spectrum (125 MHz) of purified notoamide X in CD3OD. Figure S9: DEPT135 spectrum (125 MHz) of notoamide X in CD3OD. Figure S10: 1H-1H COSY spectrum (500 MHz) of notoamide X in CD3OD. Figure S11: HSQC spectrum of notoamide X in CD3OD. Figure S12: HMBC spectrum of notoamide X in CD3OD. Figure S13: NOESY spectrum of Compound 1 in CD3OD. Figure S14: ^15^N-labeled cyclo(Pro-Trp) detected in the LC-MS analysis of the bacterial cultivation added with stable ^15^N-labeled L-proline and L-tryptophan. Figure S15: ^15^N-labeled notoamides detected in the UHPLC-HRMS analysis of the fungal cultivation added with the corresponding bacterial broth containing ^15^N-labeled cyclo(pro-trp). Table S1: ^1^H NMR (500 MHz) and ^13^C NMR (125 MHz) Spectroscopic Data of notoamide X. Table S2: Mass intensities of notoamide-type metabolites detected in the UHPLC-HRMS analysis of both the fungal mono-cultures and fungal-bacterial co-cultures.
