# Supplementary material for: Comparative Metabolomics Reveals Fungal Conversion of Co-Existing Bacterial Metabolites within a Synthetic Aspergillus-Streptomyces Community
Source: Mar Drugs. 2021 Sep 19;19(9):526. doi: 10.3390/md19090526 (PMC8472691; doi:10.3390/md19090526)
Supplement: Supplementary file 1 [file marinedrugs-19-00526-s001.zip › marinedrugs-1374660-supplementary.pdf]

Electronic Supplementary Information

Comparative metabolomics reveals fungal conversion of co-existing bacterial metabolites within a synthetic *Aspergillus-Streptomyces* community

Yutong Shi,[1,2] Yihan Ma[1], Jihua Wei,[1] Yichao Ge,[1] Wei Jiang,[1] Shan He,[2] Xiaodan Wu,[3] Xiaoqin Zhang[1], Bin Wu[1,*]

[1]Ocean College, Zhejiang University, Hangzhou 310058, China.

[2] Li Dak Sum Yip Yio Chin Kenneth Li Marine Biopharmaceutical Research Center, College of Food and Pharmaceutical Sciences, Ningbo University, Ningbo 315800, China

[3] Center of Analysis, Zhejiang University, Hangzhou 310058, China.

[*]Correspondence and requests for materials should be addressed to B.W. (email: wubin@zju.edu.cn; Tel./Fax: +86-580-2092258)

Table of Contents

Chemical and Reagents

Parameters for the Designed Co-culture Devices

Metabolomics Sampling and UHPLC-ESI-HR-MS Analysis

Data Processing and Multivariate Data analysis

Detailed Parameter for Molecular Networking on GNPS

Structure Elucidation of the Co-Culture-Induced Notoamide X

List of Figures and Tables

Figure S1. Establishment of a previously-described stationary co-culture device for culturing of multispecies strains that are physically separated but can exchange small molecules

Figure S2. A synthetic fungal-bacterial community composed of *Aspergillus sclerotiorum* DX9 an*d Streptomyces* sp. WU20 generated in a co-culture device.

Figure S3. Molecular networking of the samples derived from the co-culture and the corresponding fungal monoculture.

Figure S4. OPLS-DA analysis for the metabolic comparison of Pro-Trp-added co-cultivations and untreated co-cultivations.

Figure S5. ^{1}H NMR spectrum (500 MHz) of purified cyclo(Pro-Trp) in CD_3OD.

Figure S6. ^{13}C NMR spectrum (125 MHz) of purified cyclo(Pro-Trp) in CD_3OD.

Figure S7. ^{1}H NMR spectrum (500 MHz) of purified notoamide X in CD_3OD.

Figure S8. ^{13}C NMR spectrum (125 MHz) of purified notoamide X in CD_3OD.

Figure S9. DEPT135 spectrum (125 MHz) of notoamide X in CD_3OD.

Figure S10. ^{1}H-^{1}H COSY spectrum (500 MHz) of notoamide X in CD_3OD.

Figure S11. HSQC spectrum of notoamide X in CD_3OD.

Figure S12. HMBC spectrum of notoamide X in CD_3OD.

Figure S13. NOESY spectrum of Compound **1** in CD_3OD.

Figure S14. ^{15}N-labeled cyclo(Pro-Trp) detected in the LC-MS analysis of the bacterial cultivation added with stable ^{15}N-labeled L-proline and L-tryptophan.

Figure S15. ^{15}N-labeled notoamides detected in the UHPLC-HRMS analysis of the fungal

cultivation added with the corresponding bacterial broth containing ^{15}N-labeled cyclo(pro-trp)

Table S1. ^{1}H NMR (500 MHz) and ^{13}C NMR (125 MHz) Spectroscopic Data of notoamide X

Table S2. Mass intensities of notoamide-type metabolites detected in the UHPLC-HRMS analysis of both the fungal mono-cultures and fungal-bacterial co-cultures.

Chemical and Reagents

The Reagents for cultivation and extraction were purchased from Sinopharm Chemical Reagent Company, China. The organic solvents used in chromatographic separation were of analytical grade purchased from Sayfo Technology, Tianjin, China and chromatographic grade in HPLC analysis purchased from Tedia, USA. Deionized water was prepared by Reverse osmosis Milli-Q water (18MΩ) (Millipore, Bedford, MA, USA) used for all solutions and dilutions.

Parameters for the Designed Co-culture Devices

The co-culture setup (Ref. 1) consists of a conical flask (2L), a dialysis bag with sealing tools, a mechanical spring, a magnet, and a sampling rope (Fig. S1). MD77 dialysis bags (Union Carbide, Danbury, USA) were used in this research. The diameter of the dialysis bag and the mechanical spring were 4.5 cm and 4 cm, respectively. The length of the dialysis bag and the mechanical spring were 15 cm and 10 cm, respectively. All these components are easily commercially available. The dialysis bag with a molecular-weight cutoff of 8 to 14 kDa can serve to separate two culturing spaces. A mechanical spring inside the dialysis bag attracted by one piece of magnet outside the conical flask serves as a brace. Removing the magnet and then raising the rope tied to the dialysis bag can help sample the culture inside the dialysis bag. Conical flask, dialysis bag,

spring, sealing elements, and sampling rope can be autoclaved separately and assembled under a sterile hood.

Metabolomics Sampling and UHPLC-ESI-HR-MS Analysis

The culture broth (200 ml) was extracted three times with 200 ml of ethyl acetate. The EtOAc was removed under vacuum at 40 °C and a 2-mL amount of CH_3OH was added to the freeze-dried sample. Subsequently, the mixture was vortexed for 10 s and sonicated for 20 min at a frequency of 42 kHz using an Ultrasonicator 5510E-MT (Branson, Danbury, CT, USA), followed by centrifugation. The supernatant (1.5 mL) was transferred to a UHPLC vial for metabolomics analysis. The sample components were separated on a Waters Acquity ethylene-bridged hybrid (BEH) C18 column (2.1 × 150 mm, 1.7 μm) using a gradient of 20-100% CH_3OH in H_2O over 10 min and holding for 5 min at 100% CH_3OH with a flow rate of 0.6 mL/min. The column was kept at 40 °C, and 5.00 μL of the extracts was injected. Mass spectrometric detection was performed with a quadrupole-TOF MS (TripleTOF 5600, AB Sciex, Concord, Canada). Data was acquired in centroid mode with source/fragmentor voltage of 120 V, both negative and positive mode ion detection between 150-1500 *m*/*z* at a scan time of 0.2 seconds, gas temperature of 250 °C, and capillary voltage of 4,000 V. Leucine-enkapheline was infused through the reference probe for internal calibration during data acquisition.

Data Processing and Multivariate Data analysis

The HR-MS data were analyzed using Analyst® TF 1.6 and Peakview 2.1 (AB SCIEX). Markers between 150 and 1500 Da were collected with an intensity threshold of 10k counts and retention time and mass windows of 0.2 min and 0.1 Da, respectively. The noise level was set to 100, and the raw data were deisotoped. Statistical analysis of the data was done using SIMCA (version 14.0, Umetrics, Umea, Sweden). The markers from the extracts were compared through PCA, and the data were scaled using Pareto. Two and two groups of extracts were compared using orthogonal projections to latent structures with discriminant analysis (OPLS-DA).

Detailed Parameter for Molecular Networking on GNPS

A molecular network was created using the online workflow at GNPS. The data was filtered by removing all MS/MS peaks within +/- 17 Da of the precursor m/z. MS/MS spectra were window filtered by choosing only the top 6 peaks in the +/- 50Da window throughout the spectrum. The data was then clustered with MS-Cluster with a parent mass tolerance of 0.05 Da and a MS/MS fragment ion tolerance of 0.1 Da to create consensus spectra. Further, concensus spectra that contained less than 1 spectra were discarded. A network was then created where edges were filtered to have a cosine score above 0.5 and more than 4 matched peaks. Further edges between two nodes were kept in the network if and only if each of the nodes appeared in each other's respective top 10 most similar nodes. The spectra in the network were then searched against GNPS' spectral libraries. The library spectra were filtered in the same manner as the input data. All matches kept between network spectra and library spectra were required to have a

score above 0.6 and at least 4 matched peaks. The complete molecular networking output and parameters can be accessed via https://gnps.ucsd.edu/ProteoSAFe/status.jsp?task=03c0510fb39f445ba9fb19a08d021b76

Structure Elucidation of the Co-Culture-Induced Notoamide X

Notoamide X was isolated as a white powder. The molecular formula was determined to be $C_{12}H_7NO_4$ by analysis of the HR-TOF-MS ion peak at *m/z* 252.0277 $[M + Na]^+$ (calcd. 252.0267). The ^1H and ^{13}C NMR spectra of **1** (**Table s1**) showed similar chemical shifts and the same multiplicities for 28 carbon atoms of six rings A-F as compared to the known compound notoamide K [Ref. 2] with two evident differences in the jucture site between ring C and D, indicating that nitroamide X supposedly possesses the basic structure of a nitroamide fused ring system with a modification imposed upon ring A and D. The diagnostic HMBC correlations from the proton H-21 to the two juncture carbons between ring C and D, namely the oxygenated C-3 and the di-aminated C-2 bridged these two carbon atoms with an addition ring, which generated a novel skeleton and new fused ring system that is hitherto unreported. This inference also was supported by the HR-TOF-MS results and indicates the presence of an additional ring in the molecule of notoamide X. The NOESY cross peak of δ2.54 (H-10)/δ0.87 (H-23) indicated a β-oriented H-10 (δ2.54) and the same orientation for the new ring on C-2 and C-3. The identification of an α-oriented H-11 was deduced from the analysis of the HOESY correlation between the α-oriented H-10

(δ2.74) and H-11 (δ4.71). The determination of OH-17 was inferred from the biosynthetic origin of all the nitroamide, which was confirmed by the distinctive downfield ^{13}C NMR chemical shift at δ 91.6 [Ref. 3]. According to the established data (Figure S7-S13, Table S1), notoamide X was identified as a new notoamide derivative containing a distinctive ring saddled at the juncture of ring C and D. The complete NMR signal assignments are listed in Table S1.

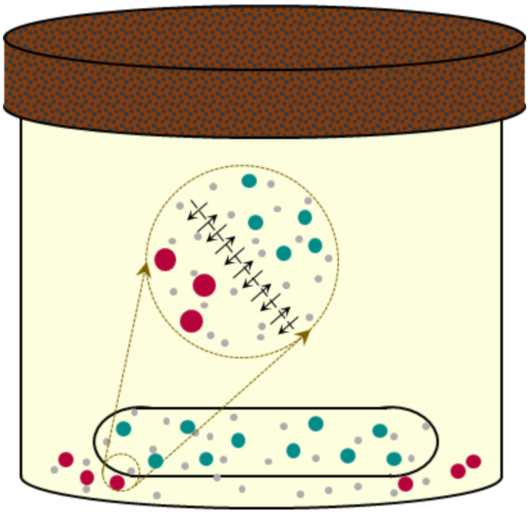

Figure S1. Establishment of a previously-described stationary co-culture device for culturing of multispecies strains that are physically separated but can exchange small molecules (Ref. 1).

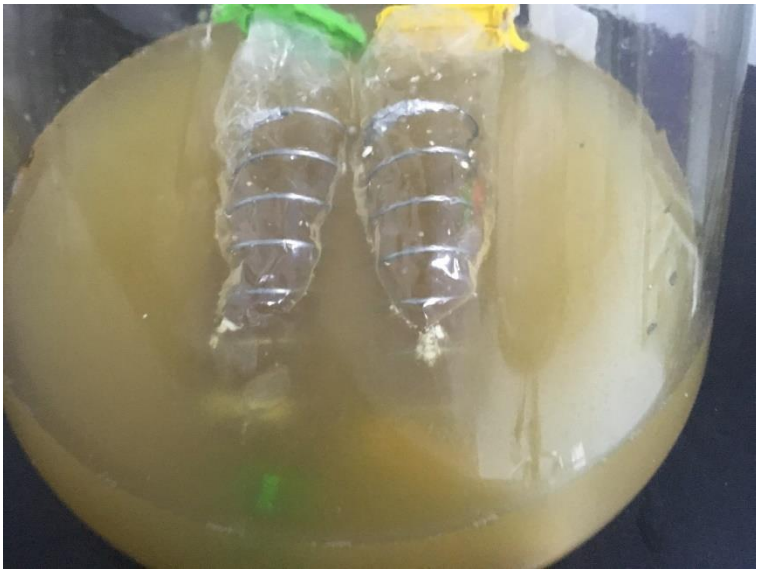

Figure S2. A synthetic fungal-bacterial community composed of *Aspergillus sclerotiorum* DX9 and *Streptomyces* sp. WU20 generated in a co-culture device.

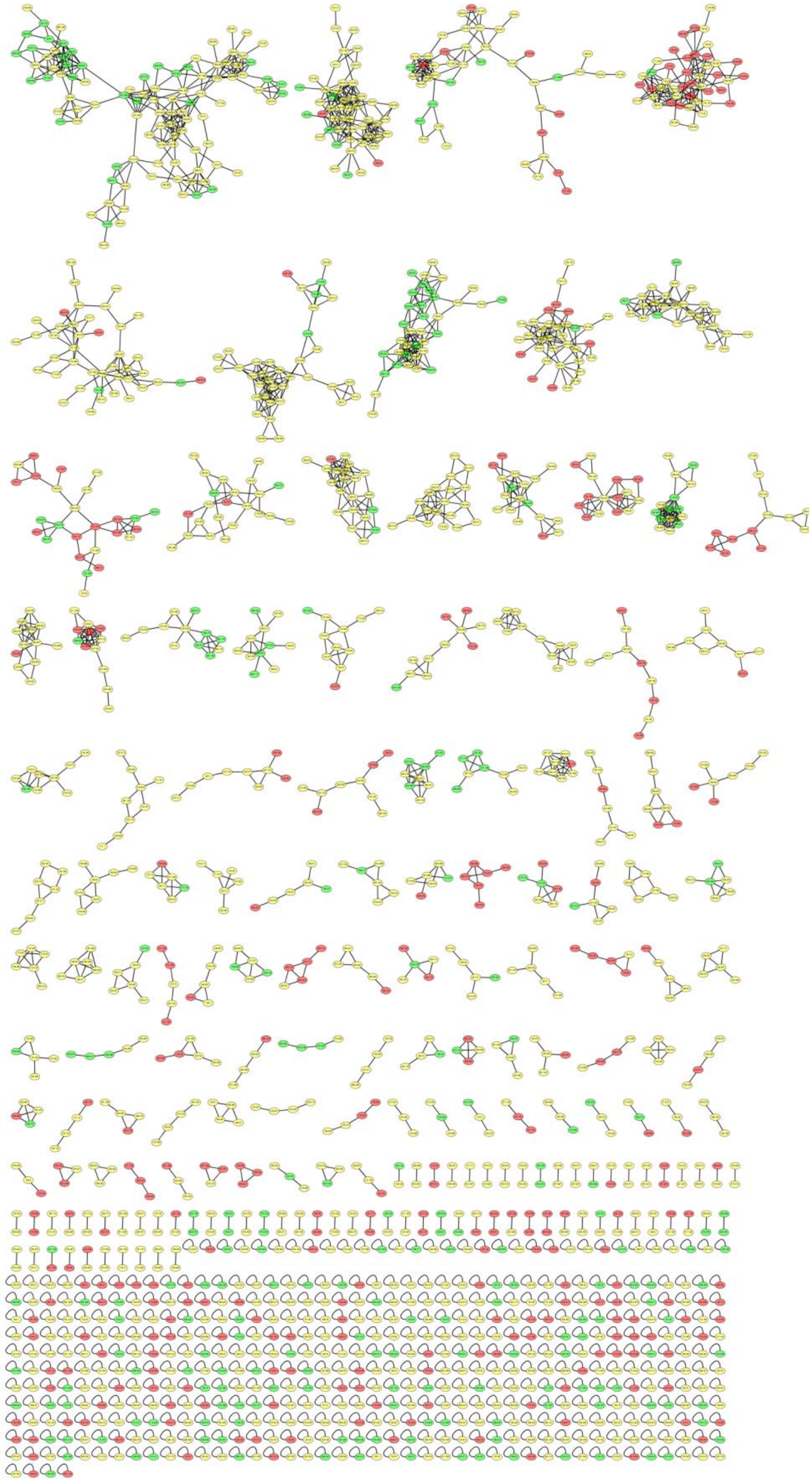

Figure S3. Molecular networking of the samples derived from the co-culture and the corresponding fungal monoculture. Red nodes were markers only presenting in the co-culture and green nodes were only detected in the monoculture. Yellow features were detected in both groups.

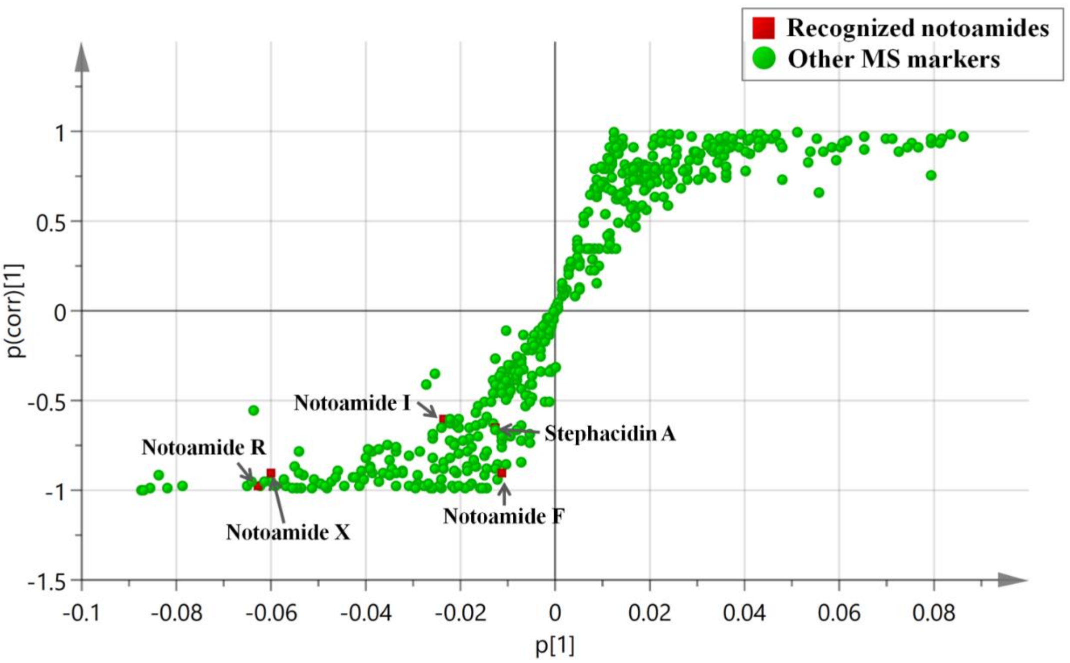

Figure S4. OPLS-DA analysis for the metabolic comparison of Pro-Trp-added co-cultivations and untreated co-cultivations.

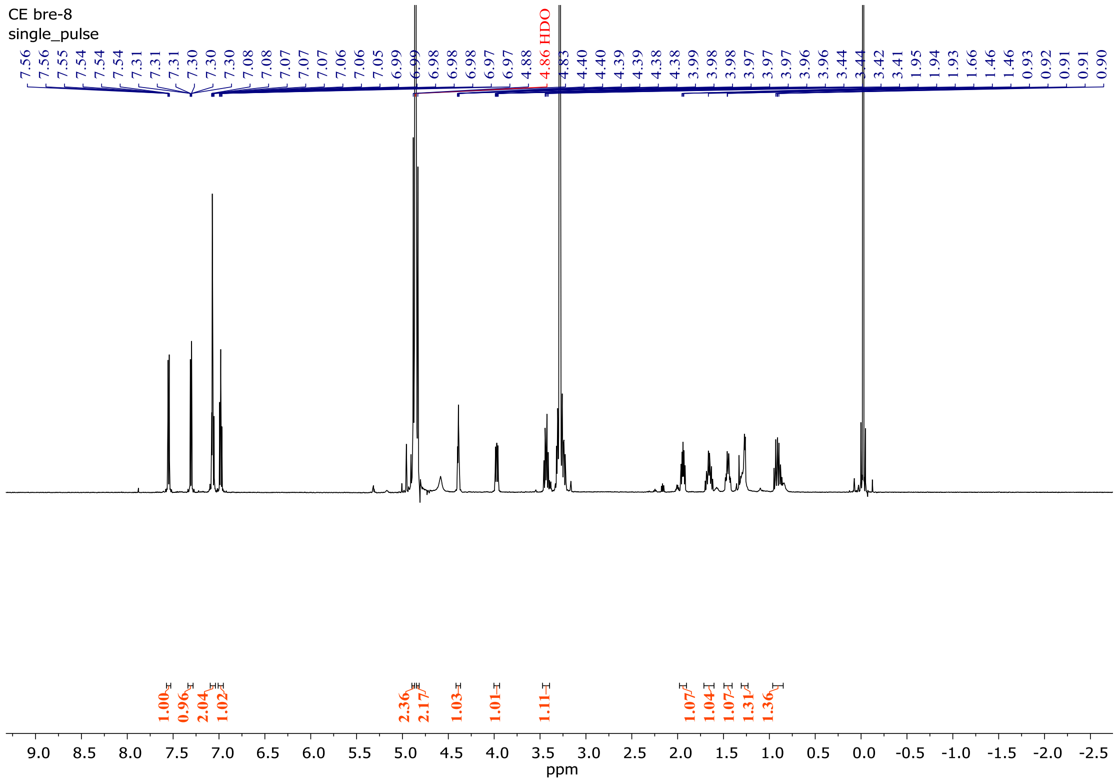

Figure S5. ^{1}H NMR spectrum (500 MHz) of purified cyclo(Pro-Trp) in CD_3OD.

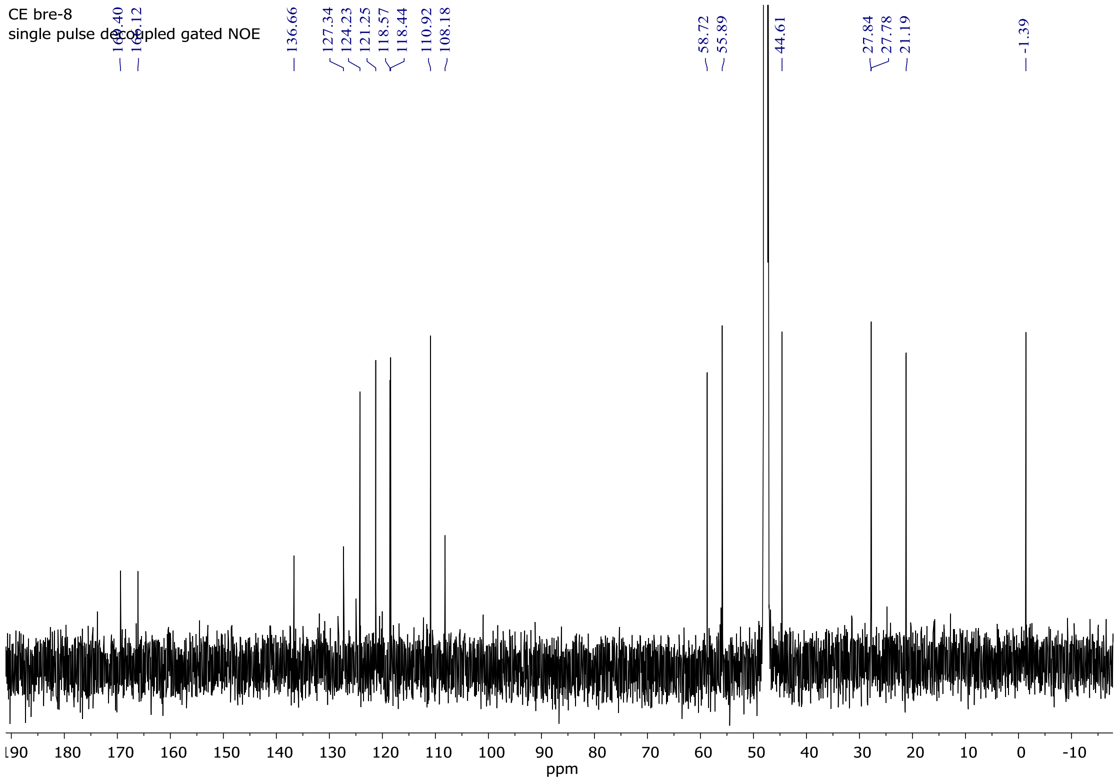

Figure S6. ^{13}C NMR spectrum (125 MHz) of purified cyclo(Pro-Trp) in CD_3OD.

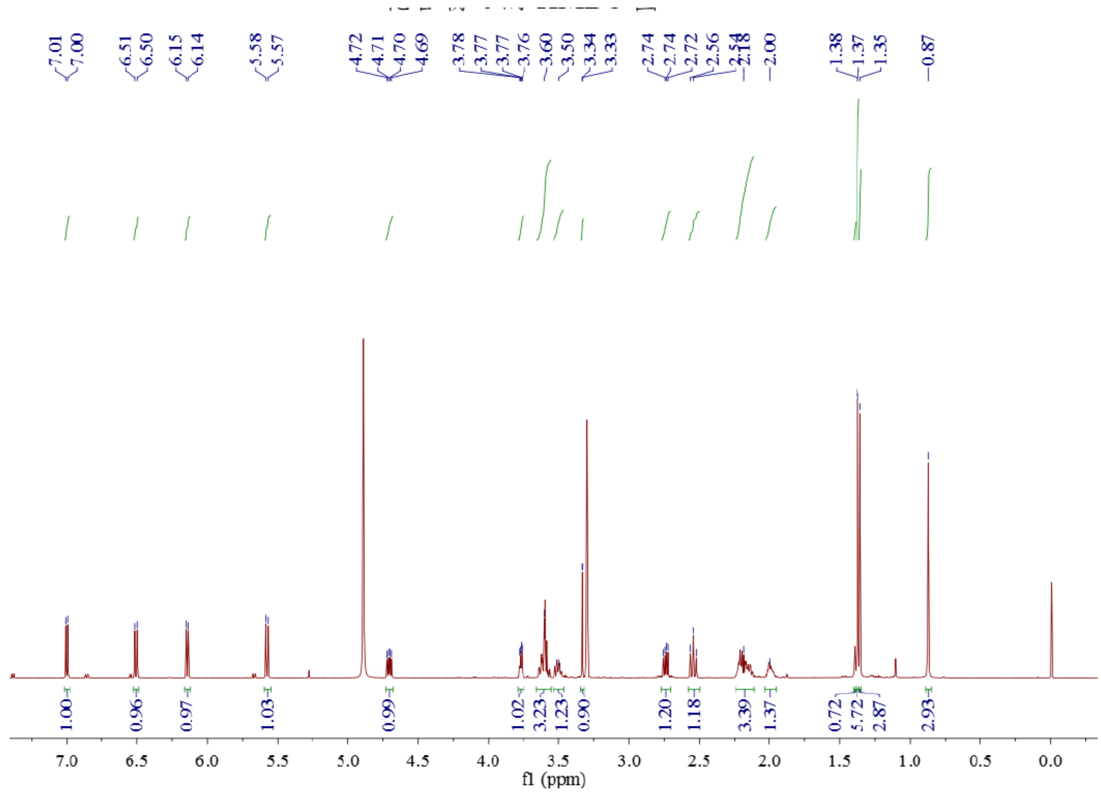

Figure S7. ^{1}H NMR spectrum (500 MHz) of purified notoamide X in CD_3OD.

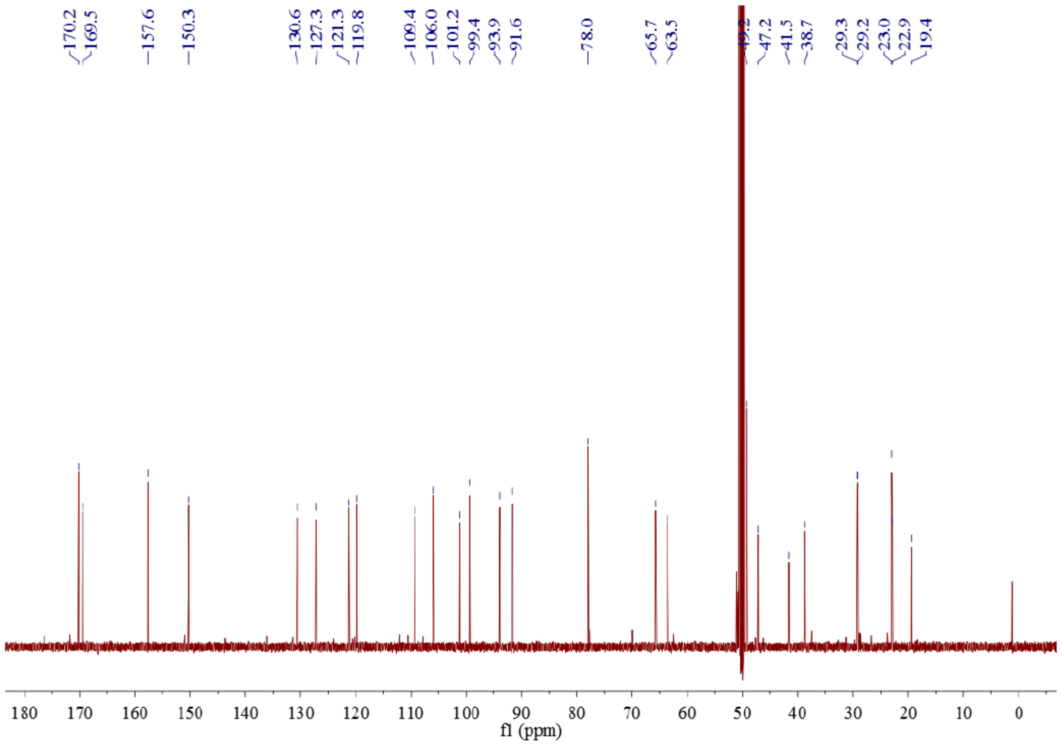

Figure S8. ^{13}C NMR spectrum (125 MHz) of purified notoamide X in CD_3OD.

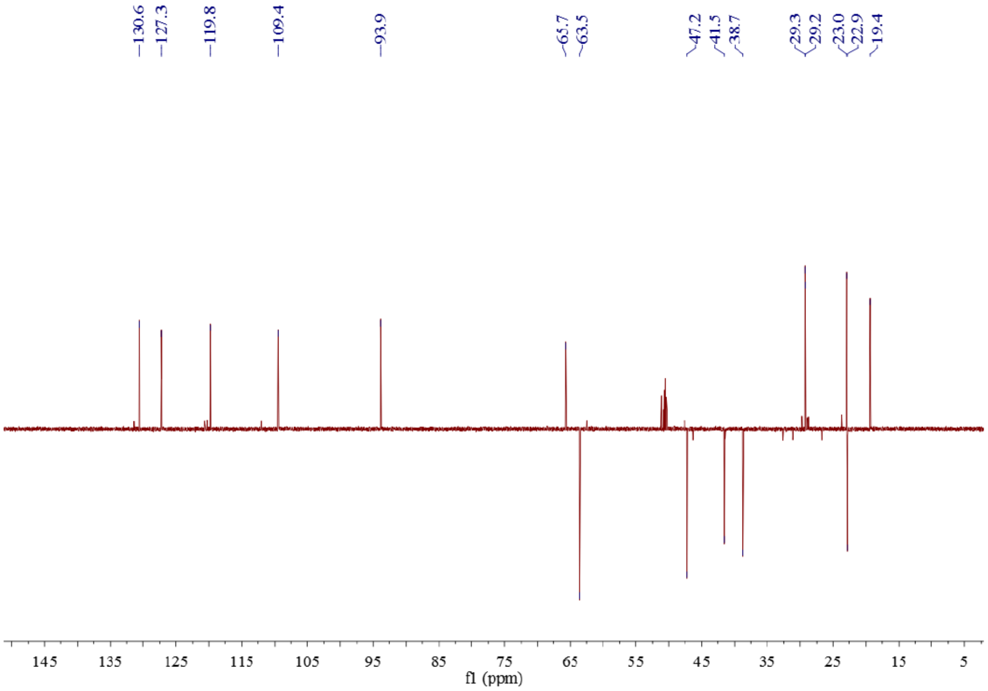

Figure S9. DEPT135 spectrum (125 MHz) of notoamide X in CD_3OD.

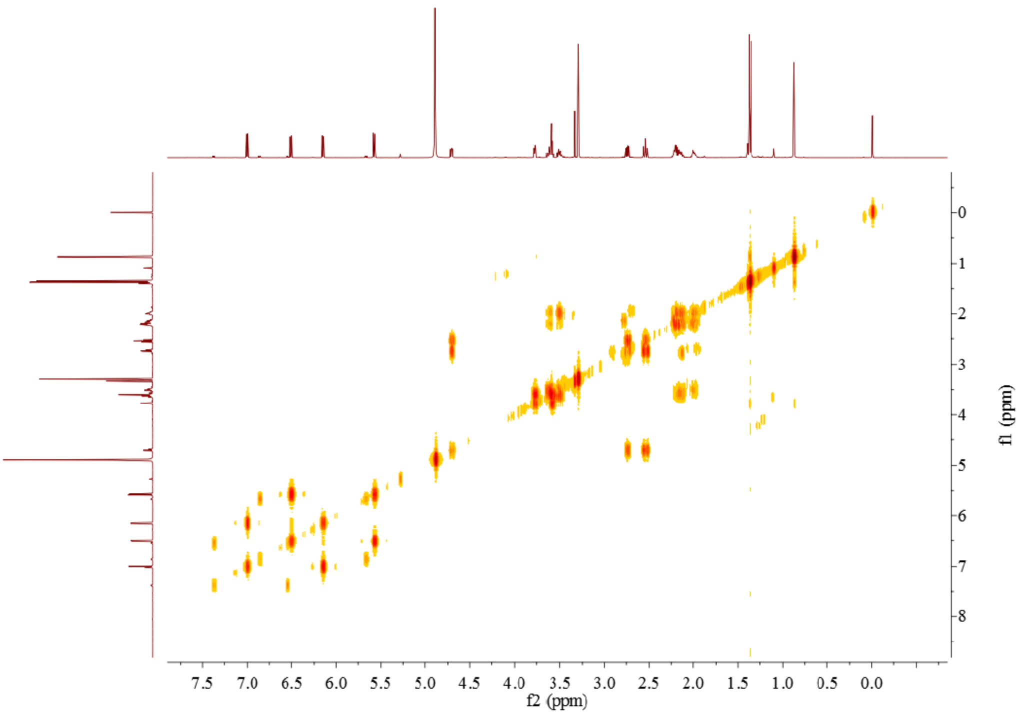

Figure S10. ^1H-^1H COSY spectrum (500 MHz) of notoamide X in CD_3OD.

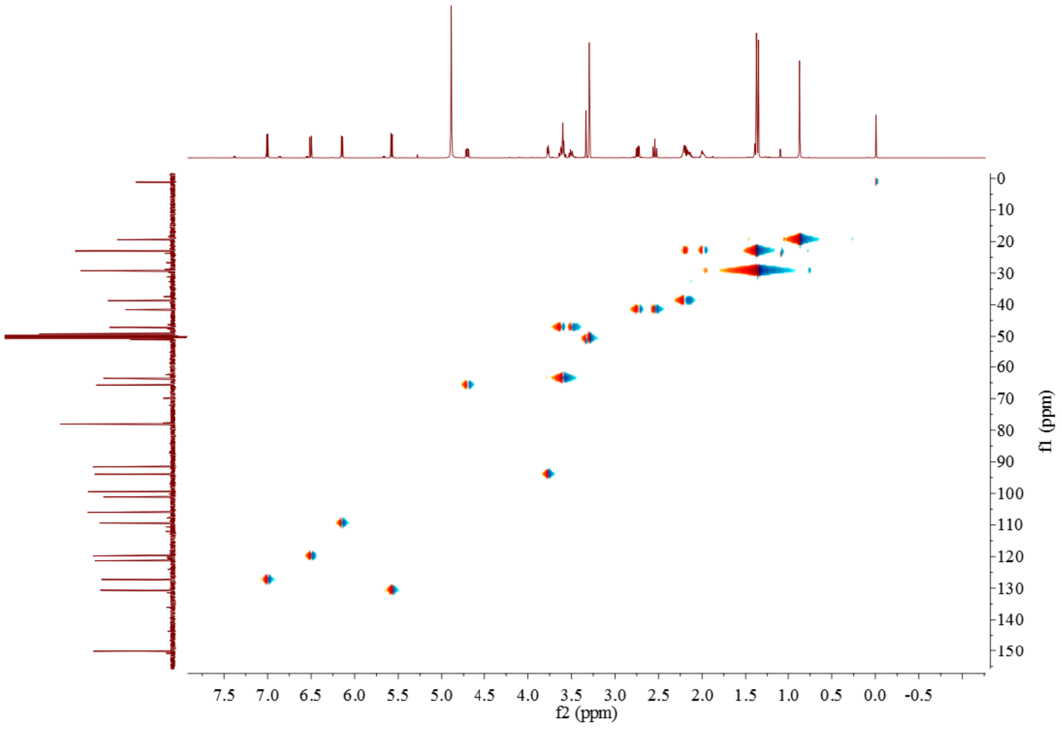

Figure S11. HSQC spectrum of notoamide X in CD_3OD.

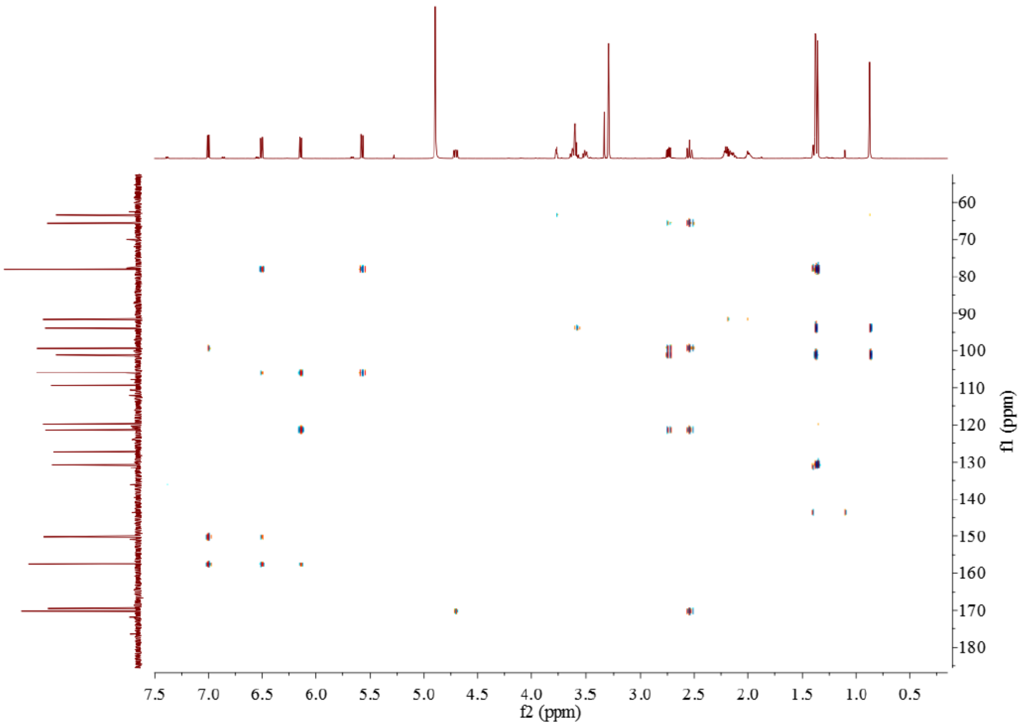

Figure S12. HMBC spectrum of notoamide X in CD_3OD.

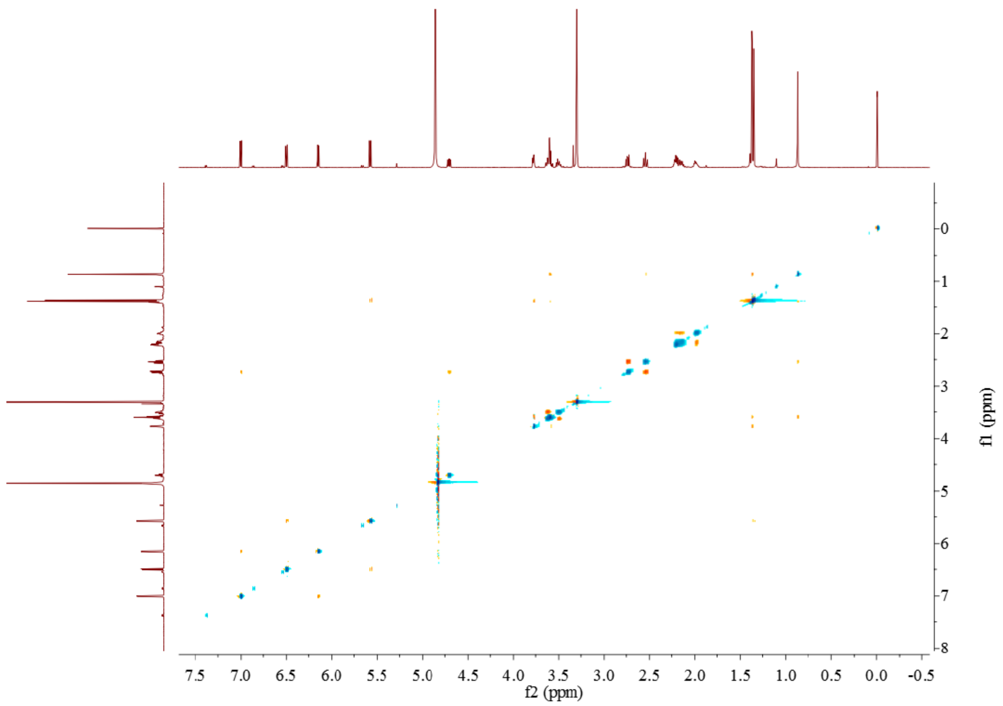

Figure S13. NOESY spectrum of notoamide X in CD_3OD.

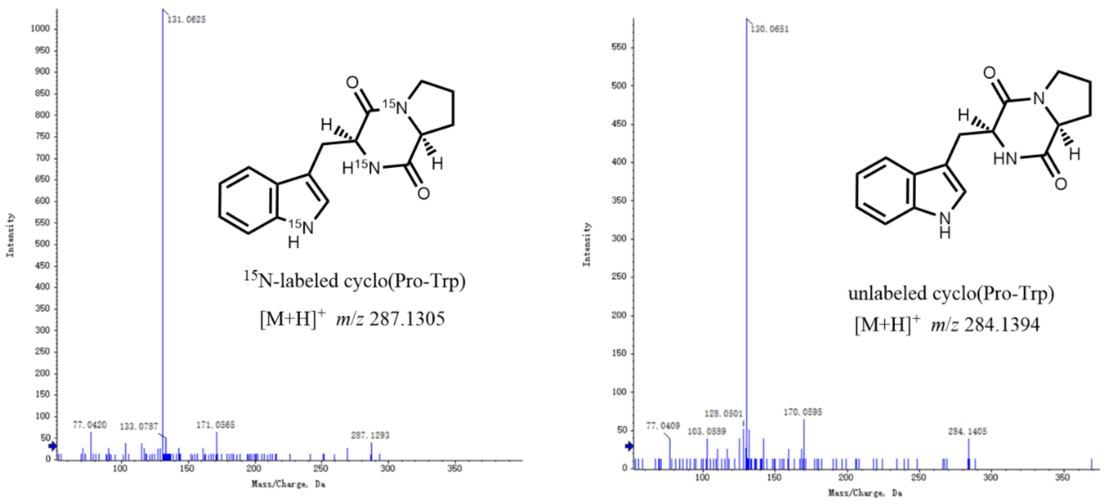

Figure S14. ^{15}N-labeled cyclo(Pro-Trp) detected in the LC-MS analysis of the bacterial cultivation added with stable ^{15}N-labeled L-proline and L-tryptophan.

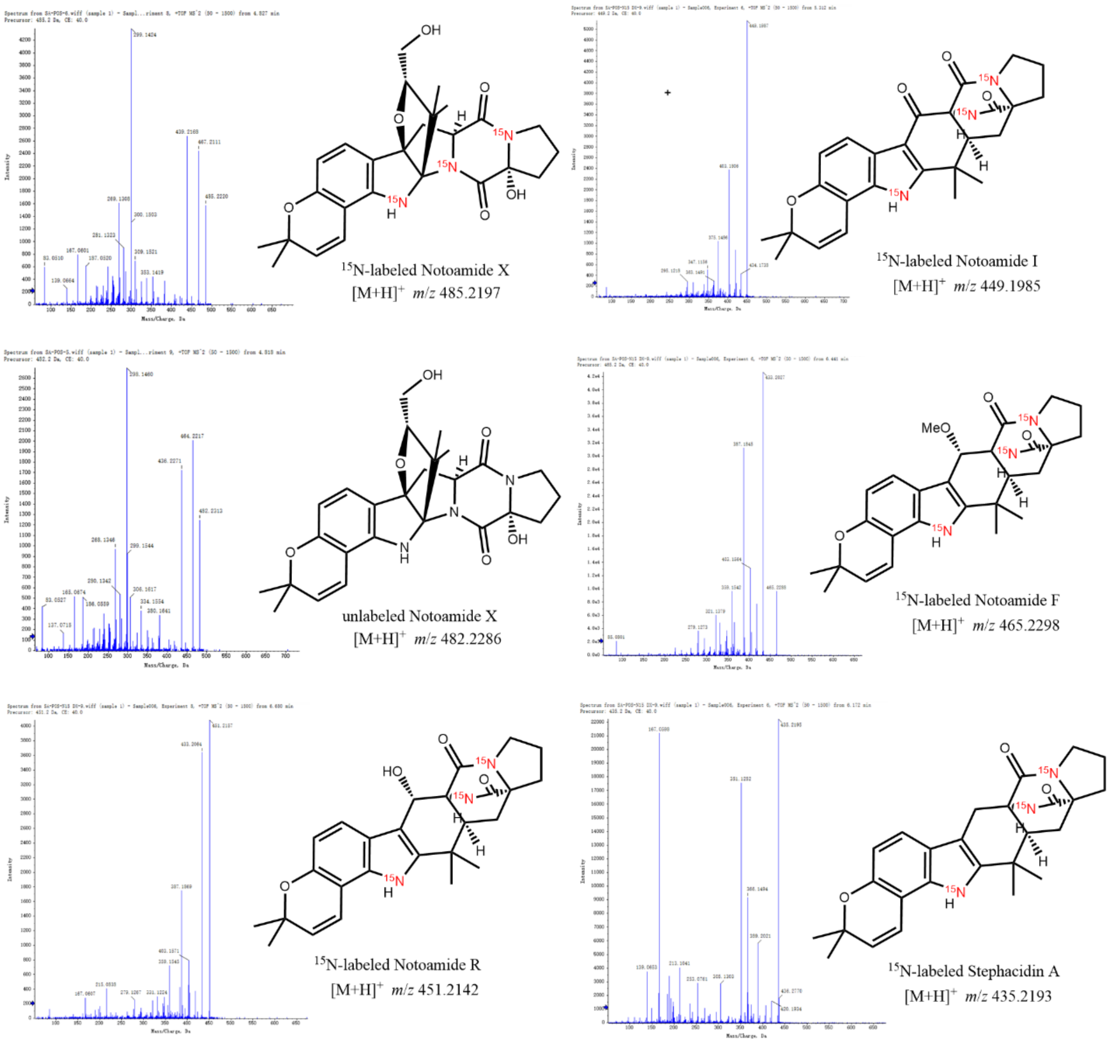

Figure S15. ^{15}N-labeled notoamides detected in the LC-MS analysis of the fungal cultivation added with the corresponding bacterial broth containing ^{15}N-labeled cyclo(pro-trp)

Table S1. ^{1}H NMR (500 MHz) and ^{13}C NMR (125 MHz) Spectroscopic Data of notoamide X (δ in ppm)

no.	δ_C^{a}	δ_H^{a}
2	101.2 C	
3	99.4 C	
4	127.3 CH	7.00 (d, 8.1)
5	109.4 CH	6.15 (d, 8.1)
6	157.6 C	
7	106.0 C	
8	150.3 C	
9	121.3 C	
10	41.5 CH_2	2.74 (dd, 12.7, 6.9)
		2.54 (t, 12.4)
11	65.7 CH	4.71 (dd, 12.0, 6.9)
12	170.2 C	
14	47.2 CH_2	3.58 (m) 3.51 (m)
15	22.9 CH_2	2.20 (m) 2.00 (m)
16	38.7 CH_2	2.18 (m)
17	91.6 C	6.48, s
18	169.5 C	
20	63.5 CH_2	3.58 (m)
21	93.9 CH	3.77 (dd, 7.1, 3.4)
22	49.2 C	
23	19.4 CH_3	0.87(s)
24	23.0 CH_3	1.37(s)
25	119.8 CH	6.51 (d, 9.9)
26	130.6 CH	5.58 (d, 9.9)
27	78.0 C	
28	29.2CH_3	1.38(s)
29	29.3 CH_3	1.35(s)

^{a}Recorded in MeOH-d_4.

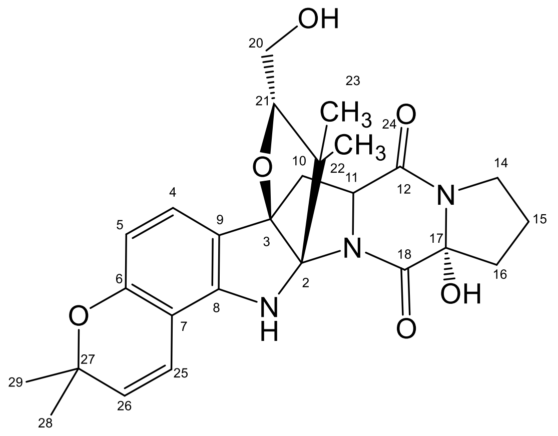

notoamide X

Table S2. Mass intensities of notoamide-type metabolites detected in the UHPLC-HRMS analysis of both the mono-cultures and co-cultures.

Compound	$[M+H]^+$/Da	RT/min	Mass Intensity					
			Mono-cultures			Co-cultures		
notoamide R	448.2231	4.2	75662	69881	80912	479166	398271	458219
notoamide X	482.2286	4.4	42604	44857	60192	139382	144884	152910
notoamide F	462.2387	5.4	31955	35102	39180	64073	72351	70295
notoamide I	446.2074	5.2	44089	45992	32101	105298	156902	65061
notoamide B	448.2231	5.8	18734	27381	22939	23912	26524	29873
stephacidin A	432.2282	5.4	21019	23017	14291	30298	35921	31558

References

1. Shi Y, Pan C, Wang K, Chen X, Wu X, Chen CTA *et al.* Synthetic multispecies microbial communities reveals shifts in secondary metabolism and facilitates cryptic natural product discovery. *Environ Microbiol* 2017; 19: 3606-3618.
2. Tsukamoto S, Kato H, Samizo M, Nojiri Y, Onuki H, Hirota H, Ohta, T. Notoamides F-K, Prenylated Indole Alkaloids Isolated from a Marine-Derived *Aspergillus* sp. *J Nat Prod* 2008; 71(12): 2064-2067.
3. Tsukamoto S, Kawabata T, Kato H, Greshock TJ, Hirota H, Ohta T, Williams RM. Isolation of antipodal (-)-versicolamide B and notoamides L-N from a marine-derived *Aspergillus* sp. Org Lett 2009; 11(6):1297-300.
